# Exérèse curative d'une tumeur stromale extra gastro intestinale (EGIST) localement avancée et fistulisée dans le grêle après traitement par Imatinib: à propos d'un cas

**DOI:** 10.11604/pamj.2014.17.247.4150

**Published:** 2014-04-01

**Authors:** Abdesslam Bouassria, Ouadii Mouaqit, Elbachir Benjelloun, Hicham Elbouhaddouti, Abdelmalek Ousadden, Khalid Mazaz, Khalid Ait Taleb

**Affiliations:** 1 Service de Chirurgie Viscérale, CHU Hassan II, Faculté de Médecine et de Pharmacie de Fès, Université Sidi Mohamed Ben Abdellah, Fès, Maroc

**Keywords:** Tumeur stromale extra-gastro-intestinale, imatinib, chirurgie curative, traitement préopératoire

## Abstract

Les tumeurs stromales extra-gastro-intestinales (les EGIST) sont des tumeurs rares qui se développent en dehors du tractus digestif. Bien que l'exérèse curative est acceptée comme un traitement de EGIST non invasive, la stratégie thérapeutique pour EGIST localement avancées et/ou métastatiques n'a pas encore été établie. Nous rapportons un cas rare de tumeur stromale extra gastro intestinale (EGIST) localement avancée et fistulisée dans le grêle qui a pu faire l'objet d'une résection curative après traitement par l'imatinib, avec une bonne évolution post opératoire. Le traitement préopératoire avec le mésylate d’ imatinib suivie par une exérèse complète (R0) est une option thérapeutique réalisable pour guérir les EGIST invasives.

## Introduction

Les tumeurs stromales gastro-intestinales (GIST) sont des tumeurs mésenchymateuses rares qui sont généralement situés dans l′estomac ou l′intestin grêle. Moins de 5% des GIST sont situés à l′extérieur du tractus gastro-intestinal: ce sont les tumeurs stromales extra gastro intestinales (EGIST). Le traitement largement accepté pour les GIST et les EGIST est une résection complète (R0) lorsque les tumeurs ne présentent pas d′invasion ou de métastase. Les progrès récents dans les biothérapies ciblées ont conduit au développement d′agents anti-tumoraux: l′imatinib et le sunitinib, inhibiteurs de la tyrosine kinase sélectif qui ont été utilisés pour traiter les EGIST invasives et/ou métastatiques.

Nous rapportons un cas de résection complète d'EGIST invasive et fistulisée dans le grêle après traitement par Imatinib.

## Patient et observation

**Figure 1:**
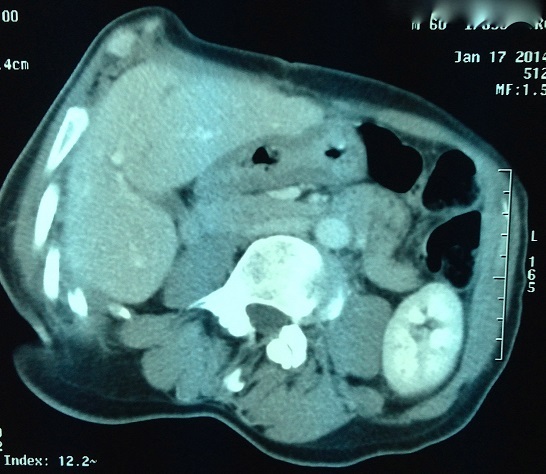
Scanner abdominal montrant la tumeur fistulisée dans le grêle

**Figure 2:**
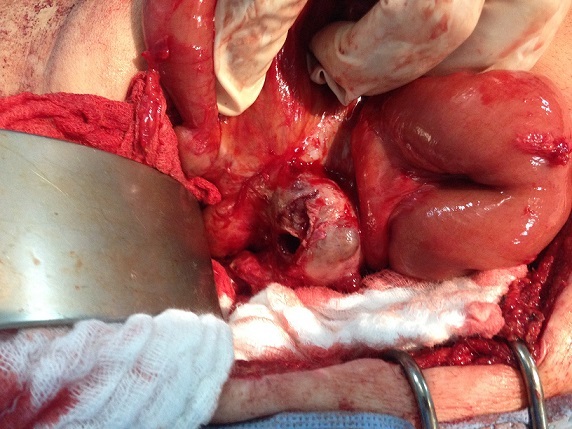
Vue per opératoire: tumeur stromale extra gastro intestinale fistulisée

**Figure 3:**
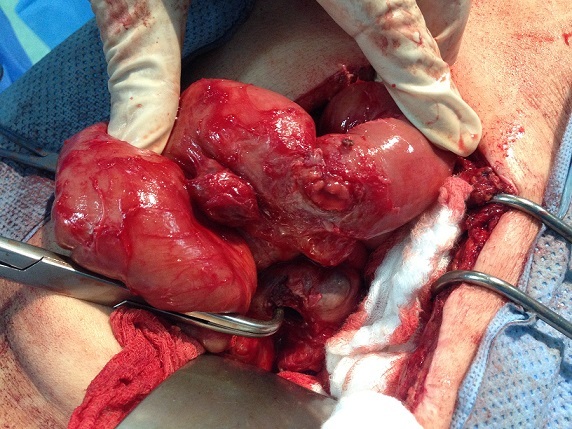
Vue per opératoire: tumeur stromale extra gastro intestinale fistulisée dans le grêle

Notre patient de 69 ans, avait bénéficié en 2007 de la résection d'une masse adjacente au carrefour iléo caecale dont l'histologie avaitrévélé une prolifération de cellules fusiformes dysplasiques qui présentaient une immunoréactivité des anticorps anti- c-kit. La masse adonc été diagnostiquée comme EGIST. En 2011, le contrôle scannographique objectivait une récidive tumorale avec une volumineuse masse mésentérique adjacente à la première anse jéjunale et une autre pelvienne (Figure 1). Dix-neuf mois après le traitement à l′Imatinib, le patient a présenté des mélénas, sans retentissement hémodynamique. Le scanner abdominal objectivait la régression de la taille des deux tumeurs, avec une fistulisation de l'une des tumeurs dans la première anse jéjunale. Vu la régression en taille des tumeurs sous traitement, la chirurgie a été proposée. Le patient était donc admis au bloc opératoire. L'exploration chirurgicale retrouvait une tumeur pelviennesans signe d'envahissement, qui a été réséquée, et une tumeur fistulisée dans la première anse jéjunale et pour laquelle une résection grêlique emportant la tumeur avec anastomose grêlo-grêlique était réalisée (Figure 2, Figure 3). Les suites post opératoires étaient simples, avec une reprise du transit a J+3. Le patient était déclaré sortant à J+5. Les résultats histologiques ont permis le diagnostic final d'EGIST dérivé du mésentère. Un traitement post opératoire à base d'imatinib a été débuté.

## Discussion

Les tumeurs stromales gastro-intestinales (GIST) représentent 85% des tumeurs mésenchymateuses du tube digestif. Ces tumeurs rares sont localisées le plus souvent au niveau de l'estomac et de l'intestin grêle[1]. Moins de 5% des GIST sont situés à l′extérieur du tractus gastro-intestinal, au niveau du mésentère, de l′épiploon et/ou du péritoine (il s'agit de tumeurs stromales extragastrointestinales: EGIST) [2, 3]. La résection chirurgicale complète en monobloc de la tumeur (résection R0) est le seul traitement potentiellement curatif des GIST et des EGIST[1, 4]. En cas de tumeur localement avancée, une exérèse large parfois mutilante n'est licite que si l'exérèse est complète, on retiendra alors l'alternative d'un traitement néo-adjuvant. Notre observation a rapporté un cas rare d'EGIST invasive, localement avancée et qui s'est finalement fistulisée dans le grêle, ayant bien répondu au traitement par l'imatinib, avec diminution considérable de la taille des tumeurs, permettant leur exérèse complète.

Si la résection complète est le traitement largement accepté pour EGIST lorsque les tumeurs ne présentent aucune invasion ou métastase vers d′autres organes [1, 4, 5], la stratégie thérapeutique pour les EGIST invasives et/ou métastatiques n′a pas encore été établie. A ce jour, la chimiothérapie avec l′imatinib et le sunitinib (inhibiteurs sélectifs de la tyrosine kinase) est généralement utilisée pour traiter EGIST localement avancées et/ou métastatiques. Pour notre patient, nous avons proposé une nouvelle stratégie impliquant la combinaison de la chimiothérapie préopératoire suivie d′une résection chirurgicale pour guérir l'EGIST localement avancée qu'il présentait. Le moment approprié pour la chirurgie après imatinib est inconnu [6]. Nous avons décidé d′effectuer une résection chirurgicale 19 mois après le traitement par imatinib mésylate, lorsque la taille des tumeurs a régressé, permettant une exérèse chirurgicale complète facile et non mutilante. Le traitement préopératoire avec le mésylate d′ imatinib suivie par une exérèse complète (R0) est une option thérapeutique réalisable pour guérir les EGIST invasives [6].

## Conclusion

Le traitement préopératoire par l'Imatinib suivi par la résection est une option réalisable pour guérir les EGIST invasives. Des études plus poussées concernant la résection chirurgicale après traitement par imatinib et portant sur un grand nombre de patients avec EGIST avancées sont nécessaires pour déterminer les indications et le « timing » de la résection chirurgicale.
